# Keeping up with the condensates: The retention, gain, and loss of nuclear membrane-less organelles

**DOI:** 10.3389/fmolb.2022.998363

**Published:** 2022-09-20

**Authors:** Emma Lacroix, Timothy E. Audas

**Affiliations:** ^1^ Department of Molecular Biology and Biochemistry, Simon Fraser University, Burnaby, BC, Canada; ^2^ Centre for Cell Biology, Development, and Disease, Simon Fraser University, Burnaby, BC, Canada

**Keywords:** nuclear condensate, membraneless organelles (MLOs), liquid-liquid phase separation (LLPS), compartmentalization, functional amyloid aggregation, eukaryota

## Abstract

In recent decades, a growing number of biomolecular condensates have been identified in eukaryotic cells. These structures form through phase separation and have been linked to a diverse array of cellular processes. While a checklist of established membrane-bound organelles is present across the eukaryotic domain, less is known about the conservation of membrane-less subcellular structures. Many of these structures can be seen throughout eukaryotes, while others are only thought to be present in metazoans or a limited subset of species. In particular, the nucleus is a hub of biomolecular condensates. Some of these subnuclear domains have been found in a broad range of organisms, which is a characteristic often attributed to essential functionality. However, this does not always appear to be the case. For example, the nucleolus is critical for ribosomal biogenesis and is present throughout the eukaryotic domain, while the Cajal bodies are believed to be similarly conserved, yet these structures are dispensable for organismal survival. Likewise, depletion of the *Drosophila melanogaster* omega speckles reduces viability, despite the apparent absence of this domain in higher eukaryotes. By reviewing primary research that has analyzed the presence of specific condensates (nucleoli, Cajal bodies, amyloid bodies, nucleolar aggresomes, nuclear speckles, nuclear paraspeckles, nuclear stress bodies, PML bodies, omega speckles, NUN bodies, mei2 dots) in a cross-section of organisms (e.g., human, mouse, *D. melanogaster*, *Caenorhabditis elegans*, yeast), we adopt a human-centric view to explore the emergence, retention, and absence of a subset of nuclear biomolecular condensates. This overview is particularly important as numerous biomolecular condensates have been linked to human disease, and their presence in additional species could unlock new and well characterized model systems for health research.

## Introduction

Life is maintained by the completion of diverse chemical reactions throughout the cellular milieu. However, relative to the immense volume of the cell, even the largest biomolecules would only come together infrequently by random diffusion. This problem is exacerbated by the size of many molecular complexes, which can require the stepwise recruitment of multiple proteins to the reaction sites (He and Weintraub, 1998; Tardiff and Rosbash, 2006; Zhou et al., 2019). This fundamental problem has been a critical driver of cellular organization, as strategies seem to have evolved to by-pass this diffusion limit. The separation of prokaryotic and eukaryotic life forms is in part founded on the principle of compartmentalization. Membrane-bound organelles are much more abundant in eukaryotes species, and they optimize reaction kinetics by grouping functional processes within distinct cellular compartments. This limits the need for biomolecules to diffuse randomly throughout the cell, by micro-concentrating effectors and targets together to enhance the reaction kinetics. Membrane-bound organelles can also provide an environment that is effectively secluded from the rest of the cell, where conditions can be optimized for specific reactions. For example, if not for the physical barriers that surround the lysosome and peroxisome, the low pH or oxidative conditions present within these structures would be incredibly harmful to other cellular components. Although cellular compartmentalization has primarily been associated with membrane-bound structures, membrane-less organelles known as biomolecular condensates have also been shown to organize biological molecules into highly dynamic molecular assemblies through the process of phase separation (Fox et al., 2002; Lamond and Spector, 2003; Brangwynne et al., 2009; Hernandez-Verdun, 2011; Nott et al., 2015; Gomes & Shorter, 2019). Here, the steady-state residency time of specific molecules are increased within one subcellular location, promoting the transition of these factors to a different phase than the surrounding environment. Once formed, the constituents of these biomolecular condensates can be exchanged with the cellular milieu, providing some significant advantages over their membrane-bound counterparts. Notably, the formation of phase separated structures can be extremely rapid (Brangwynne et al., 2009; Chujo & Hirose, 2017; Davis et al., 2019). This allows condensates to quickly respond to changing environmental conditions, which is highlighted by the role of many membrane-less organelles in stress-response pathways (Audas et al., 2016; Alberti and Hyman, 2021; Deonarine et al., 2021; McCluggage and Fox, 2021). The absence of a membrane separating the condensate constituents from the cellular milieu also allows for rapid exchange of macromolecules between these structures and the surrounding environment, which allows cells to buffer molecule concentrations in response to various stimuli (Iarovaia et al., 2019; Hasenson and Shav-Tal, 2020). While the presence of membrane-bound organelles is a defining feature of eukaryotic cells, membrane-less structures have been reported in numerous prokaryotes (Kerfeld and Erbilgin, 2015; Maccready et al., 2018; Azaldegui et al., 2021). This observation suggests that membrane-less organelles are the ancestral method of biomolecular organization, and a more thorough examination of their evolution and conservation is long overdue.

Biomolecular condensates are composed of at least two types of molecules: scaffolds and clients. Scaffolding molecules are essential components for condensate assembly, and can be protein or nucleic acid-based in nature (Banani et al., 2016, 2017). Recent interest in the non-protein-coding regions of the genome has uncovered a variety of transcripts that can serve as “architectural RNAs” for membrane-less organelle formation (Chujo et al., 2016). These long noncoding RNAs (lncRNAs) appear to function as the condensates basic framework, and possess high valency for client binding (Garcia-Jove Navarro et al., 2019; Chen and Mayr, 2022). Client molecules are condensate constituents that are not structurally essential, and often have at least two functional domains. One domain is usually responsible for associating with multivalent scaffold molecules, which facilitate the recruitment of client molecules at a specific subcellular location. The second domain is frequently intrinsically disordered and forms additional interactions with other cellular factors (Mitrea and Kriwacki, 2016). These associations give rise to dense interaction networks, which promote the binding of specific molecules, while excluding the interaction of others. Together, this represents a micro-concentration event within a defined area and drives the liquid-liquid phase separation process that gives rise to membrane-less sub-cellular structures (Chujo and Hirose, 2017; Garcia-Jove Navarro et al., 2019; Zhang and Lai, 2020). Despite many biomolecular condensates possessing dynamic and liquid-like properties, a sub-population of condensates can enter a solid-like state (Audas et al., 2016; Boke et al., 2016). In these solid structures, the condensate constituents form insoluble and immobile amyloid-like aggregates, which limit the dynamic exchange of molecules with the surrounding milieu (Woodruff et al., 2018). During the formation of these structures, there seems to be an intermediate liquid-like phase that undergoes progressive solidification through an as yet unknown mechanism (Kellermayer et al., 2008; Rambaran and Serpell, 2008; Wang et al., 2018). Regardless of the physical state of these condensates, they are capable of sequestering and consolidating specific proteins without the use of membranes, highly enrich the organizational capacity of a cell.

Based on the physical nature of biomolecular condensates, the functions of these domains are often conceptually grouped into two categories: biochemically active “assembly lines,” and more inert molecular “reservoirs.” Some suggest that the dense interaction networks within these structures can allow them to concentrate cellular factors and optimize reaction kinetics (Gall et al., 1999; Feric et al., 2016; Joseph G.). In this way, membrane-less organelles could act as molecular “assembly lines,” bringing in substrates from the cellular milieu, performing multiple reactions, and releasing the modified products, as seen in nucleoli and Cajal bodies (Hernandez-Verdun, 2005; Feric et al., 2016). The other function typical ascribed to these subcellular domains is that of a molecular “reservoir.” Here, reactions are impaired by sequestering cellular factors away from their downstream effectors, as observed in amyloid bodies and nucleolar aggresomes (Latonen et al., 2011; Audas et al., 2016). This sequestration often occurs in response to unfavorable growth conditions like thermal stress or disrupted proteostasis, with condensate assembly and disassembly regulated by stress-signaling networks (Latonen et al., 2011; Audas et al., 2016). Once the stress has subsided, these storage depots can either be disassembled to return the proteins to a functional state, or degraded to generate molecular building blocks for new protein synthesis (Yamasaki et al., 2020; Zhang and Lai, 2020; Lei et al., 2021). Given the diverse array of constituents found in many membrane-less organelles (Banani et al., 2017; Alberti and Hyman, 2021), it is highly likely that the biological role of each subcellular structure is not rigidly confined to one of these conceptual categories.

Membrane-less organelles can be found throughout the cell, though the nucleus seems to possess the greatest diversity of these structures. While many membrane-bound organelles are present in the cytoplasm, the nucleus contains only membrane-less organelles as a means of compartmentalization. For the past few decades, there has been a growing interest in the study of subnuclear biomolecular condensates, yet the presence or absence of many of these domains throughout different species has not been extensively examined. Some subnuclear domains mediate processes that are essential to the survival of all eukaryotic cells (Thiry and Lafontaine, 2005), while others act very specifically to meet the needs of an organism (Yamashita et al., 1998; Prasanth et al., 2000). Therefore, exploring the emergence and retention of some of these structures throughout time could be informative in understanding their formation and functional importance to the survival of an organism. Here, we adopt a human-centric view to explore the emergence, retention, and absence of a subset of nuclear biomolecular condensates in a cross-section of organisms (e.g., Human, mouse, *D. melanogaster*, *C. elegans*, yeast). We limit our scope primarily to animals and yeast to assess model organisms suitable for research applicable to humans.

## Nuclear condensates retained throughout the eukaryotic domain

In general, most membrane-bound organelles are conserved across the eukaryotic domain. For many organelles, this evolutionary maintenance is a possible consequence of the important functionality that they provide. Like their membrane-bound counterparts, bimolecular condensates can also be broadly observed across eukaryotic organisms. However, while some highly retained structures have been attributed essential roles within the cells, other structures are not required to maintain the viability of the cell or organism. This leaves considerable room to speculate about the selection pressures that have led to the retention of these membrane-less compartments. Here, we describe a cross-section of condensates that have; 1) been found within a wide variety of species including yeast and humans and 2) have not been shown to be missing in other eukaryotic organisms. Evidence for the existence of a condensate within an organism includes the presence of marker molecules, and retained subcellular localization and function.

### Nucleoli

By far, the most well recognized nuclear biomolecular condensate is the nucleolus. This prominent membrane-less organelle was identified over 100 years ago, and its structure has been continually refined as microscopy techniques have advanced. Nucleoli form around multiple copies of the ribosomal DNA (rDNA) cassette, a genomic region that encodes the ribosomal RNA (rRNA) and an intergenic spacer (Mélèse and Xue, 1995; Scheer and Hock, 1999; Jacob et al., 2012). These condensates appear to be present within all eukaryotic organisms, and are widely known as the site of rRNA transcription and ribosome assembly (Shaw and Doonan, 2004; Hernandez-Verdun, 2005). Despite this functional conservation, the organization of the nucleolus can be notably different among species. For example, higher eukaryotes often have multiple nucleoli per cell. These roughly spherical condensates possess a tripartite organization that subdivides the structure into the fibrillar center, dense fibrillar component, and granular component (Hernandez-Verdun et al., 2010). While each sub-region has a different molecular composition and specialized function, their collaborative goal is the genesis of ribosomal subunits (Thiry and Lafontaine, 2005; Feric et al., 2016). The nucleolus of less complex animals appear structurally simpler than that of humans, possessing only a bipartite composition. These structures contain the fibrillar center and a granular component, but lack the dense fibrillar component found in more complex organisms (Hernandez-Verdun et al., 2010). A survey of nucleoli across eukaryotes revealed that the tripartite structure may have evolved in amniotes, as the bifurcated nucleolus is observed in most non-amniotic species (Thiry and Lafontaine, 2005). This evolutionary shift from a bipartite organization may be related to the length of the rDNA cassette, which is markedly increased in amniotes. It has been theorized that longer rDNA regions allow more space for nucleolar constituents to bind, inducing the formation of this newer subnucleolar compartment (Thiry and Lafontaine, 2005). Meristematic plant cells also contain the tripartite nucleolus, though with far fewer fibrillar centers, and more dense fibrillar components than the animal tripartite nucleolus (Sáez-Vásquez and Medina, 2008). The yeast nucleolus is also notably different from other eukaryotic species. Here, a unique crescent shaped structure forms around the single array of rDNA repeats on the right arm of chromosome 7, and occupies approximately a third of the nuclear volume (Oakes et al., 1998; Matos-Perdomo and Machín 2019). Moreover, the yeast nucleolus exhibits extensive attachments to the nuclear envelope, which are not seen in the analogous human condensates. Despite the differences in structure and composition of the nucleolus between eukaryotic species, the primary function in ribosomal biogenesis is retained. Considering that eukaryotic cell survival is absolutely dependent on effective ribogenesis and downstream protein production, the highly conserved nature of the nucleolus is intuitive. The shifting complexity of the nucleolar structure could be an indication of the need to optimize or enhance ribogenesis in more complex multicellular organisms, compared to their single-celled ancestors.

Regulating protein targeting is critical for nucleolar dynamics and assembly (Hernandez-Verdun, 2006, 2011; Muro et al., 2010; Iarovaia et al., 2019). No universal targeting sequence has been identified for constituent cellular factors, but several studies have suggested that basic amino acids can act as nucleolar localization signals (Scott et al., 2010; Musinova et al., 2015). The positively-charged residues in these client proteins are thought to ionically-interact with the abundant negatively-charged scaffolding rRNA (Table 1) to drive recruitment (Musinova et al., 2015). The basic composition of the nucleolar localization signal is also consistent from insects to humans, further supporting this model for regulating nucleolar protein trafficking (Martin et al., 2015). Growing evidence suggests that nucleolar targeting can also be regulated by stress signaling (Chatterjee and Fisher, 2003; Nalabothula et al., 2010; Latonen et al., 2011; Audas et al., 2012; Iarovaia et al., 2019), as the emergence of the Nucleolar Stress Response pathway has uncovered a novel function for this well-established subnuclear compartment (Mayer and Grummt, 2005; Scott and Oeffinger, 2016; Ogawa and Baserga, 2017; Iarovaia et al., 2019). Here, the nucleolar resident Nucleophosmin (B23), a pre-rRNA processing and cleavage factor, can sequester the tumor suppressor protein ARF within the nucleolus during normal growth conditions (Korgaonkar et al., 2005). In response to DNA damage, ARF is released into the nucleoplasm, where it can promote cell cycle arrest or apoptosis by binding MDM2 and activating p53 (Rubbi and Milner, 2003; Kurki et al., 2004; James et al., 2014; Russo et al., 2021). Activation of the B23/ARF/MDM2/p53 stress-signaling axis has only been observed in vertebrate species. This can likely be attributed to the poor conservation of many of the regulatory molecules, as B23, ARF, MDM2, and p53 appear to be absent in many lower eukaryotic organisms (e.g., *D. melanogaster* and *S. cerevisiae*), and plants (Table 2). Despite this lack of conservation, a nucleolar stress response pathway has been identified in the nucleolus of the plant species *Arabidospsis thaliana*, suggesting that the stress-sensing nature of the nucleolus is not limited to animals (Ohbayashi et al., 2017; Ohbayashi and Sugiyama, 2018). Here, perturbations in ribosome biogenesis due to DNA damage induce the activity of NAC family transcription factors, that seem to act similarly to p53 in regulating gene expression. Though the proteins involved share no sequence similarity and are not considered orthologous, it is notable that the nucleolus is involved in stress response pathways across such diverse species. In this way, the nucleolus can be considered an “assembly line” condensate, for its role in ribosome biogenesis, as well as a molecular “reservoir” for its role in the DNA damage response. Though nucleoli are a defining feature of eukaryotes, these structures have adopted additional functions throughout evolution. This novel stress-sensing functionality highlights that the emergence of a biomolecular condensate is not a static process, but rather that these structures may possess remarkable adaptability to meet the changing needs of increasingly complex organisms.

**TABLE 1:** A comparison of nuclear biomolecular condensates and their scaffolding molecules across eukaryotes. Percentage of alignments were generated using NCBI protein or nucleotide BLAST alignment tools.

Condensate	Conservation	Scaffold	Scaffold conservation	Function
Nucleolus	All eukaryotes	rRNA	*H. sapiens* 18S (1969 nt). G. gallus 18S (1823 nt)—96.57%. D. melanogaster 18S (1995 nt)—82.33%. S. *cerevisiae* 18S (1800 nt)—79.85%	Ribosome biogenesis (assembly line) and nucleolar stress response (reservior)
Amyloid Body	All eukaryotes	rlGSRNA	H. sapiens rlGS (1744 nt). M. musculus rlGS (3208 nt)—0%. G. gallus rlGS (1697 nt)—0%	Cellular dormancy (reservoir) and local nuclear protein synthesis (assembly line)
Cajal Body	All eukaryotes	Coilin	*H. sapiens* (576aa). *M. musculus* (573aa)—66.61%. G. *gallus* (600aa)—51.67% *X. laevis* (536aa)—36.22%. *D. melanogaster* (634aa)—0%	snRNP maturation. (assembly line)
Paraspeckles	Mammals	NEAT1	*H. sapiens* (22,743 nt). *M. musculus* (20,771 nt)—0%	Protein buffering (reservoir) and corpus luteum maturation
Nuclear speckles	Mammals. Xenopus. Drosophila	SON	*H. sapiens* (2426aa). *M. musculus* (2444aa)—82.61%. *X. tropicalis* (5561aa)—75.84%. *D. melanogaster* (874aa)—29.49%	Transcription splicing mRNA export (assembly line)
		SRRM2	*H. sapiens* (2752aa). *M. musculus* (2535aa)—77.77%. *H. laevis* (271aa)—65.5%. *D. melanogaster (165aa)*—*52%. S. cerevisiae (cwc21-135aa)*—*60.87%*	
Nuclear stress body	Primate	Satlll	*H. sapiens* (variable length). G. *gorilla* (variable length)—>89%	Pre-mRNA maturation (assembly line); intron retention
PML body	Mammals	PML	*H. sapiens* (882aa). *M. musculus* (885aa)—69.29%. G. *gallus* (806aa)—34.45%. C. *picta bellii* (817aa)—37.14%	DNA damage telomere maintenance (assembly line)
Omega speckles	Drosophila	hsrw	No *H. sapiens* homolog	hnRNP storage (reservoir)
NUN body	*C. elegans*	Unknown		Unknown
Mei2 Dot	S. *pombe*	meiRNA	No *H. sapiens* homolog	Suppressing degradation of pro-meiotic transacripts (reservoir)

**TABLE 2:** A comparison of eukaryotic client proteins of nuclear biomolecular condensates. Percentage of alignments were generated using NCBI protein or nucleotide BLAST alignment tools.

Client Protein	Condensate	Conservation
Nucleophosmin	Nucleolus	*H. sapiens* (294aa)
		G. *gallus* (294aa)—68.12%
		*D. melanogaster* (156aa)—34.94%
		S. *cerevisiae*—no homolog
ARF	Nucleolus	*H. sapiens* (133aa)
		G. *gallus* (60aa)—0%
		*D. melanogaster*- no homolog
		S. *cerevisiae*—no homolog
p53	Nucleolus, Nucleolar aggresome	*H. sapiens* (393aa)
		G. *gallus* (367aa)—52.47%
		*D. melanogaster* (334aa)—24.31%
		S. *cerevisiae*—no homolog
EIF4B	A-body	*H. sapiens* (611aa)
		G. *gallus* (596aa)—67.37%
		*D. melanogaster* (392aa)—28.77%
		S. *cerevisiae* (436aa)—32.18%
CDC73	A-body	*H. sapiens* (531aa)
		G. *gallus* (531aa)—97.74%
		*D. melanogaster* (538aa)—57.84%
		S. *cerevisiae* (393aa)—26.89%
NONO	Paraspeckles	*H. sapiens* (471aa)
		G. *gallus* (473aa)—84.02%
		*D. melanogaster*—nonA (700aa)—43.90%
		C. *elegans* (374aa)—45.16%
		S. *cerevisiae*—Pab1p (571aa)—23.81%
SFPQ	Paraspeckles	*H. sapiens* (707aa)
		G. *gallus* (615aa)—94.03%
		*D. melanogaster*—nonA (700aa)—46.50%
		S. *cerevisiae*—Hrp1p (534aa)—28.66%
HSF1	Nuclear stress body	*H. sapiens* (529aa)
		G. *gallus* (491aa)—73.89%
		*D. melanogaster* (715aa)—49 .08%
		S. *cerevisiae* (833aa)—30.60%
DAXX	PML body	*H. sapiens* (740aa)
		G. *gallus* (738aa)—40 .85%
		*D. melanogaster*—(1620aa)—26.98%
		S. *cerevisiae*—no homolog

### Amyloid bodies

Though some stressors induce minor changes in nucleolar protein trafficking, severe insults can lead to a complete remodeling of the nucleolar space. This results in the dissolution of the typical tri- or bi-partite structure and the formation of a novel biomolecular condensate called the amyloid body (A-body). Here, in response to extreme environmental stimuli (e.g., heat shock and extracellular acidosis), a diverse set of proteins are targeted to the nucleolus (Audas et al., 2012, 2016; Marijan et al., 2019). The growing protein population dislocates the ribosomal biogenesis machinery, and promotes the assembly of large fibrillar amyloid-like aggregates (Audas et al., 2016). Like many nuclear biomolecular condensates, A-body formation is dependent on a class of lncRNA that act as scaffold molecules during formation (Table 1). Environmental stressors trigger the expression of lncRNAs from the intergenic spacer region of the rDNA cassette, which remain in close proximity to their locus of origin following synthesis (Audas et al., 2012). These seeding molecules are highly enriched in dinucleotide repeats, and are not predicted to form any secondary structure (Wang et al., 2018). This disorder may enhance the surface area of the scaffolding lncRNA, providing more binding sites for A-body constituent proteins that possess inherent amyloidogenic properties. After reaching a micro-concentration threshold within this growing subnuclear domain, client proteins are thought to fibrillate, and the biogenesis of a solid-like functional amyloid aggregate can be observed (Audas et al., 2016; Wang et al., 2018). Though amyloid aggregation is primarily associated with irreversible neurodegenerative disorders, like Alzheimer’s and Parkinson’s diseases (Ross and Poirier, 2004; Gregersen et al., 2005), A-body formation is protective to the cell and rapidly reversed following the termination of stress signaling (Audas et al., 2016).

A-bodies were initially characterized in cultured human cell lines, grown under tightly controlled environmental conditions (Audas et al., 2012; Jacob et al., 2012; Audas et al., 2016). Recently, it has been shown that A-body formation can occur in cells derived from a variety of species, such as monkeys, mice, rats, chickens, fish, and flies (Lacroix et al., 2021). Within an organismal setting, A-bodies are inducible in *D. melanogaster* (flies) and *S. cerevisiae* (yeast), demonstrating that this membrane-less organelle is not an artifact of the tissue culture system, and is widespread throughout metazoans and yeast (Lacroix et al., 2021). An analysis of the scaffolding lncRNAs that regulate A-body formation showed no sequence conservation between the human, mouse, and chicken transcripts (Table 1) (Lacroix et al., 2021). However, stress signaling did result in the expression of low-complexity lncRNA critical for A-body formation, from a region directly downstream of the rRNA coding sequence in all three organisms (Lacroix et al., 2021). It would be interesting to assess rDNA cassette transcriptomic profiles across a broader range of organism exposed to A-body-inducing stimuli. Unfortunately, this is technically challenging, as the rDNA region is removed from most bioinformatics analyses. Together, the available data suggests that the genomic location and function of these scaffolding transcripts are retained throughout different species, despite the lack of conservation in their primary sequence (Table 1).

Proteomic analyses of human A-bodies has shown that these structures contain a large array of cellular constituents, including a variety of proteins that regulate the cell cycle (Audas et al., 2016; Marijan et al., 2019). Many of the client proteins are targeted to this subnuclear domain in a stress-specific manner (Marijan et al., 2019), suggesting that cells regulate protein recruitment to tailor the stress-response pathway to each stimulus. To date, no proteomic screens have been performed on A-bodies derived from other organisms, and only the cell cycle regulator CDC73 has been observed as a client within this structure from humans to *D. melanogaster* (Lacroix et al., 2021). Biologically, A-bodies formation in human cells has been demonstrated to induce a state of cellular dormancy (Audas et al., 2016) and serve as a site for local nuclear protein synthesis (Theodoridis et al., 2021), displaying the nature of both a reservoir and assembly-line condensate. The universal presence of CDC73 within A-bodies derived from humans to flies, suggests that cellular dormancy may be a conserved role for these biomolecular condensates across metazoans. However, local nuclear protein synthesis was absent in lower eukaryotes (i.e., flies and yeast) (Lacroix et al., 2021), suggesting that A-body-mediated translation is a later evolutionary feature for this structure. Comparing the sequence divergence of CDC73 and the critical local nuclear protein synthesis translation factor eIF4B (Theodoridis et al., 2021) in *D. melanogaster* potentially underlies the mechanism of this functional difference (Table 2).

The amyloid-like biophysical properties and the severe-stress-inducible nature of the A-bodies are the hallmark of this condensate, as these features are observed in all species tested from humans to yeast. Analogous to the dual functionality of the nucleolus (i.e., ribosomal biogenesis and Nucleolar Stress Response), A-bodies appear to have both conserved and adaptive cellular roles. It is likely that local nuclear protein synthesis evolved using a pre-existing A-body framework, as this unique function is not observed in lower eukaryotes. The cause for this emerging functionality can only be speculated; however, the increased complexity of this stress response pathway in progressively more complex organisms is not surprising. Nevertheless, assessing the presence or absence of this structure in more distant species (i.e., additional fungi and plants), as well as future proteomic and transcriptomic studies from organisms across the evolutionary landscape could provide important insights into the conserved/non-conserved roles and regulatory mechanisms of this domain. As a functional and frequently observed solid-like condensate, increased understanding of A-body formation, regulation, and biological relevance could be informative in our understanding of the utility of amyloid aggregation outside of a pathological setting.

### Cajal bodies

Cajal bodies were initially discovered in neurons, but have since been identified in an array of cell types across the eukaryotic domain. Functionally, these condensates are involved in the maturation of spliceosomal RNA and small nuclear ribonucleoprotein complexes (snRNPs). This subnuclear domain serves as the site of small nuclear RNA (snRNA) synthesis and processing, which is regulated by the activity of small Cajal-body-specific RNAs (Ogg and Lamond, 2002; Cioce and Lamond, 2005; Morris, 2008; Hebert, 2010). Unlike many biomolecular condensates, Cajal bodies are built around a protein scaffold that is composed of coilin, rather than a nucleotide-based scaffold (Table 1) (Tucker et al., 2001; Cioce and Lamond, 2005; Lafarga et al., 2016). These structures are also enriched in the SMN protein complex and Fam118b, which are both necessary for Cajal body function (Narayanan et al., 2004; Li et al., 2014). SMN is involved in the recruitment of cytoplasmic snRNPs, where U snRNPs are further modified before spliceosome assembly (Sleeman and Lamond, 1999; Narayanan et al., 2004). The precise function of Fam118b in Cajal bodies remains unclear. It associates with both SMN and coilin and its depletion results in disassembly of this structure, which could suggest a scaffolding role (Li et al., 2014). Although Cajal bodies are important in optimizing the assembly of essential snRNPs, these membrane-less organelles themselves are dispensable for the survival of an organism. Depletion of coilin has been shown to interrupt Cajal body formation in a cultured cell model (Almeida et al., 1998), though it only reduces viability in a knockout mouse setting (Tucker et al., 2001). The non-essential nature of this condensate could be a consequence of its function, as the role of this subnuclear structure is to enhance snRNP maturation kinetics. Here, concentration of factors necessary for snRNP production would markedly increase the molecular efficiency of an organism, but may not dictate whether the generation of these essential complexes occurred entirely.

Although Cajal bodies are not essential to the survival of mice, structures with similar properties are present in humans (Almeida et al., 1998), amphibians (Morgan et al., 2000), flies (Liu et al., 2006, 2009), yeast (Verheggen et al., 2002; Qiu et al., 2008), and a variety of plant species (Chamberland and Lafontaine, 1993; Gall, 2000). These biomolecular condensates are generally considered Cajal bodies if they contain coilin (a marker molecule), possess a subnuclear localization, and play a role in the production of mature snRNPs. Initially, a *D. melanogaster* ortholog for coilin was elusive (Table 1) and the Cajal bodies were identified by the presence of other marker molecules, such as SMN (Liu et al., 2006). However, a more intense search identified a coilin ortholog that had very small regions of sequence identity at the amino- and carboxyl-termini (Liu et al., 2009). This suggests that the terminal protein regions could be important in the organization of weak multivalent interactions that mediate Cajal body formation. To date, no coilin homolog has been found in yeast, though Cajal body-like structures have been identified. These homologous condensates are referred to as “nucleolar body,” and are believed to represent the early ancestor of a modern Cajal body (Verheggen et al., 2001, 2002). Despite the lack of coilin, over-expression of the human SMN homolog in yeast results in the targeting of this marker protein to the “nucleolar body” (Verheggen et al., 2001). Additionally, the yeast “nucleolar body” serves as the site of U3 snoRNA maturation, a role typical ascribed to Cajal bodies in mammals (Verheggen et al., 2001, 2002; Jády et al., 2003). As many eukaryotes are reliant on extensive amounts of splicing for the production of specific mRNA molecules, the retention of this domain in a variety of species is not surprising. However, it is remarkable that this biomolecular condensate displays remarkable retention despite being non-essential for organismal survival.

## Metazoan nuclear condensates

**FIGURE 1:**
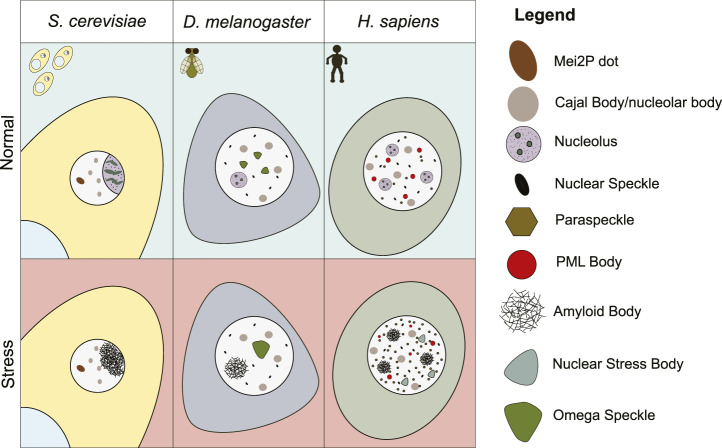
Schematic representation of a yeast, fly, and human cell in normal, or environmental stress conditions. Nuclear biomolecular condensates that are present under these conditions are depicted.

In addition to the condensates described above, there exist a variety of nuclear membrane-less organelles that are present in some metazoan species, but absent in other eukaryotes (Figure 1). This observation could highlight the correlation between more organizational and more organismal complexity, as the emergence of new cell and tissue types doubtlessly requires additional layers of cellular regulation. It is theoretically possible that metazoan-specific structures could represent another gross structural evolutionary divergence within the eukaryotic domain, akin to the adoption of membrane-bound organelles in eukaryotes versus prokaryotes. However, these “new” condensates could also represent subtler changes, or optimizations, in the cellular organization process. Some of these metazoan-specific condensates share similar features and molecular constituents with other structures observed in lower eukaryotes. Thus, these higher eukaryotic subnuclear domains may have evolved from pre-existing structures or simply sub-divided the cellular roles into two or more distinct condensates. Regardless of the mode of evolution, the analysis of metazoan-specific structures, functions, and conservation will be highly relevant to understanding the basic elements of cell biology.

### Paraspeckles

Paraspeckles were initially discovered as small dynamic structures that occupy interchromatin spaces within human nuclei (Fox et al., 2002). Recently, they have gained an abundance of attention in the scientific community, as they are an important model for examining lncRNA-protein interactions. Within this subnuclear domain, NEAT1 acts as an architectural RNA to recruit a diverse family of cellular proteins that bind along the length of the transcript (Chujo and Hirose, 2017; Fox et al., 2018; Yamazaki et al., 2018; Wang and Chen, 2020). Like nucleoli, paraspeckles possess liquid-like properties and display remarkable organization and sub-compartmentalization. Each paraspeckle has a distinct core and peripheral sub-domain, characterized by the presence of unique proteins on specific regions of the NEAT1 transcript. The 5′ and 3’ ends of this lncRNA are present in the peripheral shell-like domain (Souquere et al., 2010), while the central region of NEAT1 is localized to the condensate core, along with a group of essential proteins (e.g., NONO, SFPQ, and FUS) (West et al., 2016; Fox et al., 2018; Yamazaki et al., 2018). A variety of other non-essential client molecules have been mapped to this structure, and their biological roles have been linked to numerous cellular pathways (Masuda et al., 2015; Yamazaki and Hirose, 2015; Li et al., 2020; Wang et al., 2021). It has been proposed that formation of this structure may regulate the amount of these client proteins in the cellular milieu, potentially titrating their concentration by acting as a temporary molecular reservoir or sponge (Hirose et al., 2014). The observation that paraspeckle abundance increases in response to environmental perturbation (Mohammad Lellahi et al., 2018) could align with this functionality, as unfavorable growth conditions could lead to more proteins being “soaked-up” into this subnuclear domain to maintain homeostasis by sequestering key regulators of energy-intensive cellular pathways. In addition to regulating client protein concentrations, it has also been proposed that paraspeckles function to regulate NEAT1 availability in the cytosol (Zhang et al., 2019). Inflammatory signals promote the cytosolic release of NEAT1, where it functions to promote inflammasome activation in macrophages, suggesting that paraspeckles may also regulate aspects of the immune response (Zhang et al., 2019).

These biomolecular condensates are currently described as a mammalian-specific structure (Fox et al., 2002; Fox and Lamond, 2010; Fox et al., 2018; McCluggage and Fox, 2021); however, homologs of Paraspeckle proteins form distinct subnuclear domains in other metazoans (Table 2). For example, the *D. melanogaster* NONO homolog NonA, is a component of *Drosophila*-specific nuclear condensates, called omega speckles (Prasanth et al., 2000; Singh & Lakhotia, 2015). These foci (described below) diverge from Paraspeckles in several key ways, including the architectural RNA used and the stress-responsive nature of the domain (Singh and Lakhotia, 2015). A recent study in *C. elegans* (Pham et al., 2021), also identified NONO homolog-containing subnuclear structures, termed “paraspeckle-like” foci. Whether these nematode condensates are more closely related to the omega speckles or paraspeckles is unclear, as more rigorous examination is required. Currently, there is no literature on the existence of yeast paraspeckles, which is not unexpected considering the limited sequence-identity of potential *S. cerevisiae* homologs of NONO (Pab1p) and SFPQ (Hrp1p) (Table 2). Exogenous expression of the essential human paraspeckle protein FUS in a yeast setting resulted in the formation of cytoplasmic aggregates (Fushimi et al., 2011), further signaling the absence of this subnuclear domain in lower eukaryotes. The NEAT1 scaffolding molecule also appears to be a mammalian-specific lncRNA, thereby limiting the potential for paraspeckle formation outside of this class. Within mammals, there is no evidence of sequence conservation of the NEAT1 lncRNA transcript, suggesting that the nucleotide composition, or structure, is more important for functionality rather than the primary sequence (Table 1). An analysis of NEAT1 knockout mice has demonstrated that these paraspeckle-deficient individuals are viable, but do not develop a functional corpus luteum and fail to establish pregnancy (Nakagawa et al., 2014), implicating a potential role for paraspeckles in the mammalian-specific process of gestation. It is tempting to theorize that paraspeckles evolved to promote fertility in mammals, a functionality that would distinguish these domains further from any possible *D. melanogaster*, nematode, or yeast condensates that may be identified in the future.

### Nuclear speckles

Though they were discovered over 100 years ago (Cajal, 1910), nuclear speckles remain somewhat unclear. Many potential functions have been attributed to these dynamic and irregularly-shaped domains (e.g., transcription, splicing, and mRNA export), yet most of these roles have not been definitively established (Ihara et al., 2008; Fei et al., 2017; Hasenson and Shav-Tal, 2020). In mammalian cells, *X. laevis* oocytes, and *D. melanogaster* embryos, structures with very similar characteristics have been observed (Ségalat and Lepesant, 1992; Gall et al., 1999; Spector and Lamond, 2011), though no analogous condensates have been reported within yeast nuclei.

Nuclear speckles themselves are composed of RNA and protein constituents. Numerous poly-adenylated transcripts are found within this subnuclear domain (Fay et al., 1997; Calado and Carmo-Fonseca, 2000; Fox et al., 2002), including several low-complexity RNAs that are generated in repeat expansion disorders (Urbanek et al., 2016). The array of proteins that are targeted to these condensates are also broad, but strongly enriched in RNA-binding proteins that possess mRNA splicing and processing functionalities (Spector et al., 1991). Targeting of these resident proteins does not appear to be as efficient as that seen in other subnuclear domains, with most constituents also possessing a clear nucleoplasmic pool in addition to the nuclear speckle population (Lamond and Spector, 2003). Two proteins, SON and SRRM2, were recently found to be essential for nuclear speckle formation and organization (Ilık et al., 2020). Over evolution, both of these proteins appear to have undergone lengthening in their intrinsically disordered regions, which likely promotes the weak multivalent interactions needed to form this structure (Ilık et al., 2020). SON is absent in yeast, while SRRM2 has a significantly smaller *S. cerevisiae* homolog (Cwc21) (Table 1). Cwc21 contains the putative spliceosome-associating motif of SRRM2, but lacks the large intrinsically disordered domain that has been linked to liquid-liquid phase separation and condensate assembly (Ilık et al., 2020; Ilık and Aktaş 2021). This poor conservation further validates the absence of nuclear speckles in yeast, and suggests that the role of this structure in splicing may have evolved from a less efficient mRNA processing events that occurred within the nucleoplasm of lower eukaryotes.

### Nuclear stress bodies

Originally termed “perichromatin granules” (Mahl et al., 1989), nuclear stress bodies are stimuli-induced condensates enriched in the well-studied heat shock response protein HSF1 (Sarge et al., 1993). Under conditions of thermal or chemical stress, HSF1 activation induces the transcription of highly repetitive lncRNA from the Satellite III repeat regions present on chromosomes 9, 12, and 15 of the human genome (Denegri et al., 2002). These lncRNA range in length, from 2 to 10 kb, and following expression remain closely associated with their locus of origin (Biamonti and Vourc’h, 2010; Jolly et al., 2004; Rizzi et al., 2004). Here, the transcripts generate phase-separated nuclear stress body by acting as scaffolding molecules that concentrate stress-activated HSF1, RNA-binding proteins, and a broad family of transcription factors (Biamonti and Vourc’h, 2010). The proteomic composition of this subnuclear domain has led many to believe that this structure regulates pre-mRNA maturation (Denegri et al., 2001). However, recent next-generation sequencing results have attributed a role for nuclear stress bodies in the retention of introns by hundreds of pre-mRNA transcripts (Ninomiya et al., 2020). Mechanistically, this involves the activity of the nuclear stress body constituent SRSF9, which is modulated by post-translational modifications. This protein is phosphorylated within the structure during the recovery from thermal stress, which appears to suppress the splicing of hundreds of introns (Ninomiya et al., 2020). Altering intron retention could fine-tune gene expression and aid in the recovery from thermal stress. Despite the presence of HSF1 throughout many eukaryotic species (Table 2), the Satellite III repeats are only present in primates (Table 1), limiting nuclear stress bodies to this taxonomic order (Jarmuz et al., 2007). The late evolution of these condensates will lead to interesting speculation on the function of these structures, raising questions about whether or not nuclear stress bodies play a vital role in primate biology.

### Nucleolar aggresomes

In human cells, the formation of large nuclear and cytoplasmic aggregates, termed aggresomes, occurs when the ubiquitin-proteasome system is overwhelmed or impaired (Johnston et al., 1998; Latonen et al., 2011; Latonen 2019). These structures are enriched in poly-adenylated mRNAs and immobilize cell cycle related proteins, such as p53 (Latonen et al., 2011). Aggresomes found within the nucleus form in close proximity to nucleoli, causing a re-arrangement of the nucleolus around the new compartment. Both of these structures remain distinct, as the nucleolus retains its normal role in ribosomal biogenesis, and the aggresomes are devoid of any rRNA or nucleolar marker molecules (Latonen et al., 2011). Nucleolar aggresomes appear to act as reservoirs for misfolded proteins that have exceeded the capacity of the ubiquitin-proteasome system, as disassembly can occur following the addition of excess ubiquitin (Latonen et al., 2011). While these structures have only been observed in human cells, a similar structure has been identified in budding yeast, also in response to proteasome inhibition. These biomolecular condensates, termed intranuclear quality control compartments (INQs), form in close proximity to the nucleolus, and are also composed of misfolded proteins (Gallina et al., 2015; Miller et al., 2015). Though there are considerable similarities between the location, conditions for formation, and composition of nucleolar aggresomes and INQs, more rigorous testing will be necessary to determine if these two structures are evolutionarily related, and if nucleolar aggresomes are present beyond human cells.

### Promyelocytic leukemia bodies

The promyelocytic leukemia (PML) protein is a tumor suppressor that is associated with protein-based condensates in the nucleus called PML bodies (Melnick and Licht, 1999; Ching et al., 2005; Torok et al., 2009). Formation of these subnuclear structures is dependent on the presence of PML as a scaffolding protein, which multimerizes upon SUMOylation and recruits additional proteins, such as DAXX, SUMO-1, and Sp100 (Ishov et al., 1999; Zhong et al., 2000; Shen et al., 2006). These biomolecular condensates are not enriched in any particular nucleic acids, but they often form in close proximity to specific genes. The p53 locus is a common genetic location, and formation of PML bodies here is thought to regulate the expression of this tumor suppressor protein (Sun et al., 2003; Ching et al., 2005). Following synthesis PML bodies also play a role p53 stabilization, as the addition of post-translational modifications within this domain is believed to contribute to cell cycle inhibition (Guo et al., 2000; Conrad et al., 2016). This transcriptional and post-translational regulation of p53 has suggested that PML bodies have a tumor suppressive function, though other p53-independent roles in apoptosis, telomere maintenance, and the DNA damage response have also linked this subnuclear domain to cancer (Guo et al., 2000; Bernardi et al., 2008; Draskovic et al., 2009; Conrad et al., 2016). Unsurprisingly, PML^−/−^ mice are more susceptible to tumorigenesis, though the survival of these knockout mice demonstrates that PML, and by extension PML bodies, are not essential for organismal viability (Wang et al., 1998).

To date, PML bodies have only been observed in mammalian cells (Bernardi and Pandolfi, 2007; Batty et al., 2009). However, PML protein orthologs exist across amniotes, with varying levels of sequence and structure identity (Table 1). This could suggest that non-mammalian amniotes, like birds and turtles, also have the potential to form PML nuclear bodies, but the presence of this structure outside mammals has yet to be identified. The potential for PML body formation appears to be limited to more complex eukaryotes, as there are no identified homologs of the scaffolding protein PML (Table 1) or client molecule Daxx (Table 2) in yeast, consistent with the lack of PML body formation in the single-celled eukaryotes.

## Nuclear condensates that are not observed in humans

As described above, human cells contain a diverse array of subnuclear condensates (Figure 1). However, membrane-less organelles have been observed in various species that are not retained in humans or other higher eukaryotes. Some of these structures may have been completely lost in evolution, while others may have gradually changed their composition, structure, or function to the point where they are no longer recognizable as the same subnuclear domain. Additionally, some nuclear condensates may have been identified in only one species, and their presence in other organisms outside of humans has yet to be considered. Mapping a broader cross-section of eukaryotic organisms would be essential to track the evolutionary conservation of these domains, and could provide important information about the origins and adaptation of these unique organelles.

### Omega speckles

At the *D. melanogaster* 93D gene locus*,* the hsrω lncRNA is expressed (Lakhotia and Sharma, 1996; Lakhotia, 2011). This transcript acts as an architectural RNA that mediates the recruitment of heterogeneous nuclear ribonucleoproteins (hnRNPs) into biomolecular condensates found within the interchromatin space called omega speckles (Prasanth et al., 2000; Singh and Lakhotia, 2015). At the cellular level, these constitutive foci are present under normal environmental conditions and are thought to regulate molecular pathways by generating a reservoir of inactive hnRNPs (Prasanth et al., 2000; Singh and Lakhotia, 2015). Interestingly, heat stress can trigger the disassembly of omega speckles and the formation of a single large aggregate at the 93D locus (Singh and Lakhotia, 2015). A return to normal growth conditions causes this large aggregate to fragment, releasing smaller condensates that resemble the pre-stress omega speckles back into the nucleoplasm (Singh and Lakhotia, 2015). At the organismal level, 93D-null flies have reduced viability (Mohler and Pardue, 1984), suggesting that omega speckles may be essential for survival. However, whether the reduction in viability is due to a lack of omega speckle formation, or another function of the 93D locus is unclear.

To date, omega speckles have only been observed in *D. melanogaster*, yet these subnuclear structures bear compositional/functional similarities to the primate-specific nuclear stress bodies and client protein overlap with the mammalian-specific paraspeckle (discussed above). Regardless of the parallels between these domains, in both cases these similarities are not sufficient to consider these structures evolutionarily linked. For example, hnRNPs can be recruited to omega speckles and nuclear stress bodies by hsrω and Satellite III transcripts, respectively. Both of these scaffolding lncRNAs possess repetitive sequences, show stress-induced upregulation, and can remain in close proximity to their gene of origin following expression (Jolly and Lakhotia, 2006). Despite these similarities, there are notable differences between these structures. The Satellite III repeats are present on multiple chromosomes throughout the human genome, while hsrω transcripts are only derived from the 93D gene locus. Also, snRNPs have been found to associate with Satellite III, but not hsrω, lncRNA transcripts (Metz et al., 2004). Moreover, omega speckles are constitutively present in growing cells and altered in heat shock conditions, while nuclear stress bodies are formed only in response to stress stimulation (Sarge et al., 1993; Prasanth et al., 2000). The nuclear stress body marker molecule HSF1 also does not appear to be a component of the omega speckles formed under normal growth conditions, though it can be recruited to the large heat shock-induced aggregates that form at the 93D gene locus (Westwood et al., 1991). This makes it tempting to speculate that while nuclear stress bodies are unrelated to constitutive omega speckles, they may be a distant relative of the large aggregates that form during heat stress. Thus, these large omega speckle-like aggregates may represent a distinct biomolecular condensate, analogous to the constitutive nucleoli and stress-inducible A-bodies (Jacob et al., 2012; Audas et al., 2016; Lacroix et al., 2021). The presence of omega speckles has not been thoroughly examined outside of *D. melanogaster*, and additional data would help determine the evolutionary stage at which these structures are lost or modified.

### NUN bodies

Recently, a novel membrane-less subnuclear structure was identified by differential interference contrast microscopy in *C. elegans*, termed the NUN body (Pham et al., 2021). Here, 4–10 granular phase-separated subnuclear foci could be seen in nematode neuronal and glial cells (Pham et al., 2021). To date, there is limited information about these condensates, as no constituent molecules have been identified (Table 1). An analysis of marker proteins for a diverse array of subnuclear structures, has demonstrated that NUN bodies appear to be distinct from established nuclear domains, including nucleoli, Cajal bodies, paraspeckles, and nuclear speckles (Pham et al., 2021). The narrow cell-type specificity of NUN-bodies will be interesting to study with regard to functionality. Their exclusivity to neurons and glia could suggest that this structure has a neuronal-specific role, though more information is required. Clearly, identification of molecular constituents will be of the utmost importance in assessing the conservation and biological significance of the NUN bodies. Until this is done, it will be almost impossible to establish whether these condensates are a conserved element of neuronal and glial cell biology, or if they represent a nematode-specific domain.

### Mei2 dot

Mei2 is an RNA-binding protein in *S. pombe* (fission yeast) that is involved in meiosis (Yamashita et al., 1998). This protein functions as an essential inducer of pre-meiotic DNA synthesis and meiosis I by binding to meiRNA, a polyadenylated lncRNA transcript derived from the *sme2* gene on chromosome 2 (Yamashita et al., 1998). Similar to Satellite III (nuclear stress bodies), rIGSRNA (A-bodies), and hsrω (omega speckles) lncRNAs, once transcribed the meiRNA transcripts remain in close proximity to their locus of origin (Shimada et al., 2003). When mei2 is de-phosphorylated, its localization shifts from the cytoplasm to the nucleus, where it is able to directly bind meiRNA. Here, meiRNA appears to act as a scaffold molecule (Table 1), concentrating mei2 into a single phase-separated nuclear punctate structure called a mei2 dot. This structure also sequesters the protein Mmi1 (no human homolog), which functions to degrade pro-meiotic mRNA transcripts (Harigaya et al., 2006). The formation of this nuclear condensate appears to be essential for the induction of meiosis I, likely by suppressing Mmi1-mediated degradation of pro-meiosis transcripts (Shichino et al., 2014). As this subnuclear dot regulates the shift of a cell from mitosis to meiosis, the observation of mei2 dots only in the single-celled eukaryotes is not surprising. The transient shifts from sexual to asexual reproduction observed in yeast are not observed in multicellular species. However, future research to identify any similar structures in primordial germ cells could be interesting, as these cells also undergo a shift from mitosis to meiosis. Yet, unlike unicellular organisms, these cells do not shift back to a state of mitosis once they have undergone meiosis, until they have become fertilized. Therefore, a phase separated structure that could rapidly influence this change in reproductive state seems unlikely.

## Conclusion

Biomolecular condensation is a widely observed phenomenon within cells, and is an important method of compartmentalization within the nucleus. While the existence of a nuclear compartment is a defining feature of eukaryotes, the phase separation that organizes this structure displays remarkable diversity between species. Given the important roles that membrane-less organelles have in regulating cellular processes, it is to be expected that many types of these biomolecular condensates are dysregulated within a disease setting (Spannl et al., 2019; Sabari, 2020). Some subnuclear structures increase in abundance (Simchovitz et al., 2019), while others display altered biophysical properties like an aberrant transition from a liquid-like to a solid-like state (Mateju et al., 2017). Subtler changes in the protein composition (Kunapuli et al., 2006) or the mutation of condensate components (Yoshida et al., 2011; Simchovitz et al., 2019) have also linked nuclear condensates to disease etiology. In many cases, it is unknown whether these alterations in condensate dynamics are causative to the progression of the disease, protective, or simply a byproduct of pathogenesis. Recent studies have linked nuclear speckle dysregulation to Alzheimer’s disease, with SRRM2 miss-localized to the cytoplasm of brain tissue in Alzheimer’s disease patients (McMillan et al., 2021). Because SRRM2 (Table 1) and nuclear speckle formation appear to be present throughout metazoans, model organisms like *D. melanogaster* and *C. elegans* could be useful in studying this link to neurodegenerative disease. A-bodies also have a connection to neurological disorders, as the proteinaceous amyloid plaques seen in Alzheimer’s, Parkinson’s, and prion-based diseases share many of the biophysical properties associated with the cellular proteins found within these solid-like structures (Audas et al., 2016). As A-bodies are inducible and reversible amyloid aggregates that are observable throughout metazoans and yeast (Lacroix et al., 2021), an analysis of the formation and disassembly of this structure could provide an exciting opportunity to study critical amyloidogenic events in a number of model organisms. Some membrane-less organelles have also been linked to cancer. For example, PML bodies have been shown to regulate aspects of cell growth (Guo et al., 2000; Conrad et al., 2016), and the presence of these structures in mammals (i.e., mice) has furthered the exploration of the role of these structures in cancer. The depletion of the PML protein in a mouse model system has demonstrated that knocking out this essential scaffolding molecule increases the risk of tumorigenesis (Wang et al., 1998; Trotman et al., 2006). Whether the tumorigenic effects of this knockout mouse can be directly associated with the absence of the biomolecular condensate or an unrelated function of the PML protein are not clear. The presence of the PML protein in other amniotes (Table 1) may suggest that this structure exists in species beyond mammals, which could provide additional organismal settings to study this pathological effect.

In general, our analysis has revealed that the formation of many of nuclear structures is not essential for cell viability, but does confer some organismal viability advantages by optimizing different processes. This is clear in the formation of the mammalian paraspeckles, and PML bodies. A lack of either structure is not detrimental to the viability of the cell, but the organism is not as fit due to impaired fertility (paraspeckles) or an increased risk of tumor development (PML bodies). It appears that the formation of nuclear biomolecular condensates in general functions more to optimize important cellular reactions like splicing, mRNA processing, ribosome biogenesis, cell cycle regulation, and hnRNP assembly, rather than to enable them completely. As the need for an increased abundance of these processes occurs as species become more complex, the diversity of condensates appears to increase as well (Figure 1). Overall, understanding the evolutionary conservation of these structures will help decipher fundamental biological pathways in eukaryotic organisms and establish new model systems, with powerful genetic tools, that can study the role of biomolecular condensates in disease etiology.
